# Impact of occupational hazards on neurological health status in Pakistan: Challenges and recommendations

**DOI:** 10.1002/iid3.1180

**Published:** 2024-02-07

**Authors:** Zoaib Habib Tharwani, Yumna Salman, Maryam Irshad, Mariam Adnan Rizvi, Muhammad Hasanain, Abdullah Malikzai

**Affiliations:** ^1^ Department of Medcine Dow University of Health Science Karachi Pakistan; ^2^ Ayub Medical College Abbottabad Pakistan; ^3^ Department of Medcine Jinnah Sindh Medical University Karachi Pakistan; ^4^ Dow Medical College Karachi Pakistan; ^5^ Kabul University of Medical Sciences Kabul Afghanistan

**Keywords:** neurological health, occupational hazard

## 1 INTRODUCTION

The European Agency for Safety and Health at Work defines occupational hazards as short‐ and long‐term workplace dangers that impair the health and well‐being of employees.^1^ The Occupational Safety and Health Administration (OSHA) has classified these dangers into six categories: safety, physical, chemical, biological, ergonomic, and work organization or psychosocial hazards.^2^ The severity of occupational dangers can be assessed by statistics given by the International Labor Organization (ILO), which reports two million deaths each year as a result of workplace injuries or illnesses, 270 million and 160 million, respectively.^3^ These numbers, however, are an under‐representation of the situation as most industrial injuries and deaths go unreported.^4^

Pakistan has around 78.9 million workers, making it one of the world's largest labor forces.^5^ However, workplace safety of the workers is given negligible regard. These workers face physical and chemical hazards, as well as psychosocial risks including stress, overwork, burnout, and hostile work situations. Furthermore, a lack of education, low occupational safety awareness, shortage of safety equipment, poverty, and the absence of a suitable national system for registering occupational injuries and casualties endanger health and safety of the workers.^4^

According to the ILO, the prevalence of work‐related injuries in Pakistan is 2691 per 100,000 employees, which is significantly higher than its neighboring country China where the rate is 1891 per 100,000.^6^, ^7^ Comparison with other Asian countries is not possible due to lack of reporting of data to ILO. The industries with significantly higher rates of work‐related injuries in Pakistan were agricultural (29.3%) and construction (19.7%).^6^, ^7^ Mining, timber logging, fishing, and maintaining electrical cables are also among the high‐risk occupations. Similarly, large‐scale businesses such as textile, leather, metal, fertilizer, and cement manufacturing use toxic ingredients that also endanger workers' health and safety.

Neurological impairment is one of the significant consequences of occupational hazards among workers. Industrial exposure to substances such as lead, mercury, arsenic, and organophosphates play a significant role in the development of neuropathological diseases in developing countries.^8^ Research suggests a cause‐and‐effect relationship between the development of motor neuron diseases and industrial solvent exposure.^9^ Mechanical nerve injury can also arise as a result of the nature of job, such as handling specific instruments and executing repetitive skilled hand movements such as musicians or those working on computers for long hours.^10^, ^11^ These can range from carpal tunnel syndrome among painters and computer users to nerve damage in jobs requiring significant weight‐lifting and handling risky equipment.

This article aims to identify and highlight the impact of several occupational risk factors on the neurological health of employees in Pakistan, as well as to make recommendations to address this issue.

## 2 METHODS

We searched databases including PubMed, Scopus, and Google Scholar to look up relevant article using MeSH terms “occupational hazards,” “neurological disorders,” “sleep disorders,” and “traumatic brain injury.” Articles included fulfilled the following criteria: (i) articles in English language, (ii) full length articles, (iii) reporting at least one association of certain occupation with any neurological disorder. Articles in language other than English, abstract‐only articles, commentaries, and LTEs were excluded from this review. In addition to articles from the above‐mentioned sources, we also used reliable websites such as OSHA, ILO, World Health Organization (WHO), and so forth. News articles were only used to supplement the information used from reliable sources. Included articles were screened by two authors, and a third author was consulted in case of any discrepencies.

## 3 RESULTS

Several neurological impairments have been related to occupational hazards ranging from mild sleep disturbances to severe injuries like traumatic brain injuries (TBI), hearing loss, amyotrophic lateral sclerosis (ALS), Parkinson's disease (PD), and so on.^12^, ^13^ In Pakistan, the majority of neurological impairments caused by occupational hazards have been associated with the agriculture and construction industries, owing to a lack of proper work safety procedures and equipment unavailability.

A correlation between occupational hazards in the agricultural sector and the incidence of neurological diseases is demonstrated in a meta‐analysis by Gunnarsson et al. Exposure to pesticides and lead has been specifically found to increase the likelihood of developing conditions such as PD and ALS by a minimum of 50%.^14^ Moreover, prolonged exposure to heavy metals including Mercury (Hg), Magnesium (Mn), and Iron (Fe) can lead to the development of neuropathies, cognitive impairment, and neurodegenerative disorders.^15^

The agricultural sector requires a large quantity of water to enable the optimal growth of crops. Consequently, workers within this industry are exposed to significant levels of contaminated water, thereby exposing them to a higher risk of developing severe neurological impairments. A study conducting a comprehensive analysis gathered a total of 216 samples to compare the levels of lead (Pb) in surface water (tap water) and untreated ground water in 18 distinct districts within Pakistan. The findings showed that a large proportion of the samples, 88% (190 out of 216), had elevated levels of Pb, with mean values ranging from 13 to 135 parts per billion (ppb).^16^ This rate significantly surpasses the maximum acceptable limits of 10 ppb as suggested by the WHO.^16^ Furthermore, research has discovered that 300 million gallons per day of industrial effluent and domestic sewage discharges are mostly discharged into the Lyari and Malir rivers in Karachi, particularly in coastal regions.^17^ The majority of these contaminations originate from around 6000 companies, encompassing sectors such as chemical, textile, metal, petroleum, and oil.^17^ These industries are primarily situated in coastal regions, hence constituting the primary source of water pollution resulting from industrial leakage or accidents.

A substantial portion of Pakistan's fertile land is based in rural areas, where workers harvest vast quantities of maize, cotton, rice, and sugarcane.^18^ Approximately one‐fifth of Pakistan's GDP (gross domestic product) is generated by the agricultural sector, which is linked to detrimental effects on the neurological health of employees.^19^ Despite its economic advantage, this industry is detrimental to the neurological health of its employees. Continuous exposure to loud machinery (tractor, harvester, and irrigation equipment) and heat stress increase workers' susceptibility to progressive hearing loss. Due to a lack of manpower and personal protective equipment, many workers are exposed to low‐frequency sounds for longer periods of time, resulting in tinnitus, headaches, and eventually hearing loss.^20^ Moreover, workers in the electrical power industry are highly susceptible to electromagnetic field exposure. This exposure significantly increases the risk of developing ALS and Alzheimer's disease by 10%.^14^

Construction is another major industry associated with neurological injuries among workers. The most frequent neurological consequence for these employees is TBI, which can be difficult to manage.^13^ TBI among construction workers typically occurs secondary to falls and according to an analysis, 2210 fatal TBI cases were reported among construction workers in the United States. Considering the high incidence rate of 2.6 per 100,000 full‐time workers in a developed country, we can only imagine how high the incidence would be in a third‐world country, such as Pakistan.^21^ In addition, sleep disorders and hearing impairments are common among workers in this industry.^12^ A recent cross‐sectional study by Sathvik et al. shows a significant association between construction industry and insomnia which primarily affected sleep duration and sleep deprivation.^22^ According to reports, over 78 million Pakistanis are employed in the construction industry, exposing them to a greater risk for neurological harm.^23^

In addition, a poor legal framework, limited transparency and human resources, and a lack of awareness regarding occupational health and safety (OHS) are core factors that increase the risk of occupational hazards and eventually cause neurological illnesses. According to reports, 62 million employees are women and children who are illiterate, have a low socioeconomic background, and are semi‐skilled, thereby exposing themselves to numerous occupational hazards.^24^, ^25^ Poor infrastructure, a lack of regulations, and negligent law enforcement play a significant role in industrial catastrophes.^24^ To aid in reducing work‐related injuries and ailments, the ILO proposed a document in 2006 containing numerous OHS protocols and conventions. In contrast to many other ILO conventions, Pakistan did not ratify this one.^24^ This shows the government's profit‐driven mentality, in which output and revenue take precedence over worker health and safety. Similarly, the imperfections in the OHS law make it impossible to access and monitor the correct application of health and safety laws. As a result, many industries jeopardize the safety of their employees, and a number of difficulties arise due to a lack of accountability and transparency.^25^, ^26^ A cross‐sectional study was conducted to determine the prevalence of various neurological diseases in both rural and urban areas. According to the study, headache disorders (33.4%), nerve root lesions (27%), and stroke (72.7%−82%) are the most prevalent neurological disorders.^27^ In addition, the study analyzed 10,786 outpatients and found that, compared to urban residents, approximately three‐fourths (7828, 72.6%) of outpatients from rural hospitals suffered from ischemic stroke (72.7%) and psychiatric disorders (2.1%).^27^ This number is especially concerning in rural areas due to the limited availability of medical services, the absence of enforcement of labor laws, job insecurity, and unpaid family work.^26^

## 4 DISCUSSION

Pakistan, as a developing nation, faces various risk factors associated with the emergence of neurological diseases It is imperative to explore the association between occupational hazards and development of neurological disorders to improve work safety as well as quality of life. The type of neurological disorder differs with different occupations. Agriculture industry is found to be significantly associated with development of neurological disorders particularly PD, ALS, and Alzheimer's, due to exposure to harmful substance in pesticides and other heavy metals. Moreover, considering the lead toxicity in water supply, it is undoubtedly a significant contributor to the development of neurological deficits among workers in the agricultural industry. The reason for why agricultural industry is the most associated with neurological disorders is that majority of the rural dwellers are farmers and a huge chunk of the GDP of the country is generated by agriculture, thus, this is a very common occupation among the population putting majority of the population at a higher risk.

Construction industry is another major occupation which is associated with neurological disorders, particularly, sleep disturbances and TBI. Even when considering the risks of different occupations separately, a multitude of factors are associated with the development of neurological disorders. These mainly include problems at the administrative level, such as government involvement, lack of awareness, and lack of transparency with human resources in different occupations.

### 4.1 Efforts

Pakistan has implemented various strategies to address occupational hazards and their detrimental impacts. The country has shown a strong commitment to the 2030 Agenda for Sustainable Development, making it the first country to completely adhere to the Sustainable Development Goals. Consequently, significant advancements have been made in pursuit of sustainable development. Moreover, policies and regulations have been enacted to comply with international labor norms, further improving OHS measures. These collective endeavors have yielded notable progress toward creating a hazard‐free work environment.^28^

The CLEAR cotton project, which was implemented from March 2018 to February 2023, serves as an admirable illustration of efforts aimed at eliminating the widespread occurrence of occupational hazards. The collaborative initiative between the European Union and the ILO played a significant role in eradicating child labor within the cotton production sector and mitigating the risks associated with the use of hazardous pesticides in the cotton fields located in Southern Punjab. The objective was accomplished by the implementation of a community awareness campaign regarding the detrimental consequences associated with pesticide usage, coupled with the provision of OHS training programmes aimed at fostering compliance with established safety protocols within the working environment. Furthermore, the families at‐risk were provided training to earn through an alternate means.^29^

Furthermore, the establishment of Pakistan workplace Health and Safety Act of 2018, as well as the Centre for Environmental and Occupational Health by the National Institutes of Health in Pakistan, serve as concrete evidence of the government's commitment to addressing the adverse consequences associated with workplace hazards. Despite the recognition of various initiatives, there is a substantial requirement for further efforts to alleviate the incidence of occupational and neurological injuries in Pakistan.^30^, ^31^

### 4.2 Recommendations

According to the ILO, the yearly mortality rate of two million can be attributed to human activity and is therefore preventable, rendering these fatalities both avoidable and potentially eliminable.^32^ In Pakistan, industries such as agriculture, mining, and fishing play a major role in generating revenue, accounting for approximately 50% of the country's GDP.19 However, these occupations also pose significant risks, resulting in fatalities and injuries. Thus, it is imperative to identify and implement effective measures to establish safe working environments in Pakistan and effectively address OHS concerns.

First, it is evident that the field of Occupational Medicine in Pakistan is currently experiencing a significant lack of attention and inadequate implementation. Occupational risks are associated with several risk factors, each necessitating specific treatment and preventive measures. These measures can only be effectively identified during the early stages by specialists in this field. Furthermore, the timely identification of a condition can significantly alleviates the financial costs associated with it. According to a survey, the medical cost for a single injured worker amounts to 32,000 Pakistani rupees ($112). With Considering the current conditions of inflation in Pakistan, it is anticipated that this expense will show a substantial increase in the near future.^33^ There exist compelling reasons to advocate for the promotion of occupational medicine. Consequently, the implementation of routine health checks for employees will expedite the shift from a curative approach to a preventive one by promptly detecting initial indications of neurological diseases.

Another aspect that should be considered is the extended duration of work, which, as indicated by the ILO, constitutes a significant risk factor resulting in approximately 750,000 fatalities per year.^32^ A systematic review conducted with the aim of finding out the relation between prolonged working hours and cognitive impairment demonstrated that arduous extended work hours can significantly diminish cognitive function and pose substantial hazards for workers engaged in safety‐sensitive tasks, such as operating heavy machinery.^34^ To optimize productivity, it is imperative that workers are not exposed to excessively long working hours and are provided adequate intervals of rest between shifts. To ensure the well‐being of workers, both mentally and physically, it is essential to establish and enforce regulations and guidelines that prioritize their safety. Furthermore, regular surveys should be conducted to identify potential sources of exposure, and a reporting system should be implemented to enable workers to report any suspected instances of exposure. Noncompliance with these measures should be met with strict disciplinary action.

In contemporary society, the utilization of social media platforms and news channels has emerged as a means to effectively demonstrate the severe repercussions and potential dangers associated with workplace hazards. Additionally, they have the ability to spread information to a vast audience regarding strategies that can be implemented to mitigate these issues. Moreover, the government might actively engage in reinforcing exercises in various private and public institutions, aimed at educating staff members on the significance of safety protocols and disaster management. Furthermore, to alleviate the dissemination of neurotoxic exposure in occupational settings, several additional strategies can be implemented, which encompass the promotion of protective equipment utilization, enhancement of ventilation systems, guaranteeing appropriate disposal of hazardous waste, and offering regular training sessions to workers regarding the appropriate handling of hazardous substances.

Lastly, it is imperative to establish rapid response healthcare teams in areas that comprise of industries most susceptible to occupational hazards, with the aim of delivering immediate and quality first aid and essential healthcare services to effectively address the ongoing issue.

All these recommendations can be implemented through a socioecological model which would require an interplay of individual, interpersonal, community, organizational, and societal factors. This can further be broken down into education and training, health screening, and stress management at an individual level. Interpersonal factors would involve a better workplace and peer support system. Community and organizational factors include health programs, easy healthcare access, OHS polices, and ergonomic practices. Lastly, societal factors coupled with media and communications aspect of awareness may provide a more structured plan which can be implemented in a simple way.

## 5 CONCLUSION

In conclusion, it is crucial to address the implications of occupational hazards on neurological health within the context of Pakistan. The current literature highlights the various hazards encountered by workers across industries including agriculture and construction. Persistent challenges in the field of occupational medicine include the continuing issues of extended working hours and inadequate governmental frameworks. The recommendations include several key strategies, such as implementing regular health examinations, the establishment of methods to monitor and control working hours, and the use of social media platforms and educational initiatives to enhance public awareness. It is imperative to prioritize the improvement of safety measures, waste disposal practises, and the provision of timely medical aid. Through these steps, Pakistan has the potential to improve workers' safety and well‐being.

## AUTHOR CONTRIBUTIONS

**Zoaib Habib Tharwani**: Conceptualization and project administration. **Yumna Salman**: Writing—original draft of manuscript. **Maryam Irshad**: Writing—original draft of manuscript. **Mariam Adnan Rizvi**: Writing—original draft of manuscript. **Muhammad Hasanain**: Writing—reviewing and editing the manuscript. Abdullah Malikzai made the critical comments and revision. All authors revised and approved the final draft.

## CONFLICT OF INTEREST STATEMENT

The authors declare no conflict of interest.
